# Heart failure‐related elevation of carbohydrate antigen 125 identified by pre‐operative cardiopulmonary exercise testing

**DOI:** 10.1002/anr3.70023

**Published:** 2025-08-27

**Authors:** R. G. Davies, F. Fiorini, D. M. Bailey

**Affiliations:** ^1^ Department of Anaesthesia University Hospital of Wales Cardiff UK; ^2^ Neurovascular Research Laboratory, Faculty of Life Sciences and Education University of South Wales Pontypridd UK

**Keywords:** CA125, exercise test, heart failure, ovarian neoplasms

## Abstract

Heart failure is a major peri‐operative risk factor associated with significant postoperative morbidity and mortality. Traditional biomarkers used in heart failure management include natriuretic peptides. Carbohydrate antigen 125 biomarker is well known to be elevated in ovarian cancer but can also be elevated in heart failure, particularly right‐sided heart failure and heart failure with preserved ejection fraction. We report the management of a 71‐year‐old woman with a presumed diagnosis of ovarian cancer based on imaging and an elevated carbohydrate antigen 125, who underwent cardiopulmonary exercise testing as part of the pre‐operative assessment. Exercise testing, despite being sub‐maximal, identified significant but asymptomatic heart failure. Surgery was deferred and cardiology‐led optimisation resulted in normalisation of her carbohydrate antigen biomarker, refuting her ovarian cancer diagnosis and avoidance of a major intra‐abdominal surgery. This case highlights the role of biomarkers, such as carbohydrate antigen 125, in heart failure treatment and the use of exercise testing in heart failure diagnosis and management. Cardiopulmonary exercise testing facilitated the identification of hidden comorbidities leading to better pre‐operative risk stratification, optimisation and collaborative decision making.

## Introduction

Carbohydrate antigen 125 (CA125) is commonly associated with ovarian cancer and can be used to monitor recurrence and response to treatment, but is less well known for its role in heart failure management [1]. Heart failure is a significant peri‐operative risk factor associated with high mortality and morbidity [2]. Pre‐operative diagnosis of heart failure may present opportunities for optimisation before surgery.

Subjective assessment of peri‐operative risk in elective surgery is considered to perform poorly at predicting postoperative outcomes. Cardiopulmonary exercise testing (CPET) is an incremental exercise test on a cycle ergometer in which a patient follows a standardised protocol until symptom limitation. The test assesses the cardiovascular, respiratory and musculoskeletal responses to exercise and can be diagnostic and prognostic, even if the test is sub‐maximal. Cardiopulmonary exercise testing is well established in the evaluation of heart failure severity, prognosis and treatment [3]. Cardiopulmonary exercise testing is frequently used in pre‐operative assessment clinics before major surgery for assessing known comorbidities, identification of hidden comorbidities, risk stratification, guiding optimisation and facilitating shared decision making. It is considered to be the gold standard assessment of functional capacity [4]. Exercise intolerance in patients diagnosed with cancer is common and patients living with cancer exhibit marked impairments in oxygen uptake, particularly patients receiving neoadjuvant cancer therapies or undergoing surgery. This makes CPET an ideal tool to assess a patient's cardiorespiratory fitness. Peak oxygen uptake ($\dot{V}O_{2}$ peak), anaerobic threshold and ventilatory equivalent for carbon dioxide $\left(\dot{V}E/\dot{V}{\mathrm{CO}}_{2}\right)$ are the CPET measurements commonly associated with outcomes in heart failure and peri‐operative risk, but there are other CPET metrics which are pathognomonic in heart failure.

We report the management of a patient with elevated CA125 and suspected ovarian cancer who was diagnosed with significant heart failure following exercise testing in our pre‐operative assessment clinic and after optimisation avoided high‐risk surgery.

## Report

A 71‐year‐old woman (weight 55 kg; height 165 cm; BMI 20.2 kg.m^−2^) attended our pre‐operative assessment clinic for CPET and pre‐operative risk assessment. She had been diagnosed with probable ovarian cancer and was scheduled for laparotomy, oophorectomy and omentectomy. She had a right‐sided 10 cm adnexal mass together with elevated CA125 biomarker (92 kU.l^−1^, normal range 0–35 kU.l^−1^) and Risk of Malignancy Index Calculator score 828 (> 200 is high risk for malignancy). She had been under surveillance for the previous 4 years with an asymptomatic right‐sided adnexal cyst and normal CA125 levels. She had atrial fibrillation, a previous stroke, chronic obstructive pulmonary disease, trigeminal neuralgia and depression. She denied dyspnoea on exertion and was able to climb two flights of stairs without breathlessness. Current medications included bisoprolol, furosemide, enalapril, apixaban, carbamazepine, citalopram and salbutamol. Six weeks before her pre‐operative assessment, she was hospitalised with a diagnosis of atrial fibrillation and heart failure. During this admission, she had bilateral pleural effusions on chest radiograph and her echocardiogram demonstrated severe left ventricular systolic dysfunction (ejection fraction 34%), impaired right ventricular function and moderate mitral regurgitation. Repeat chest radiograph, performed 2 weeks before her pre‐operative assessment, was normal.

**Figure 1:**
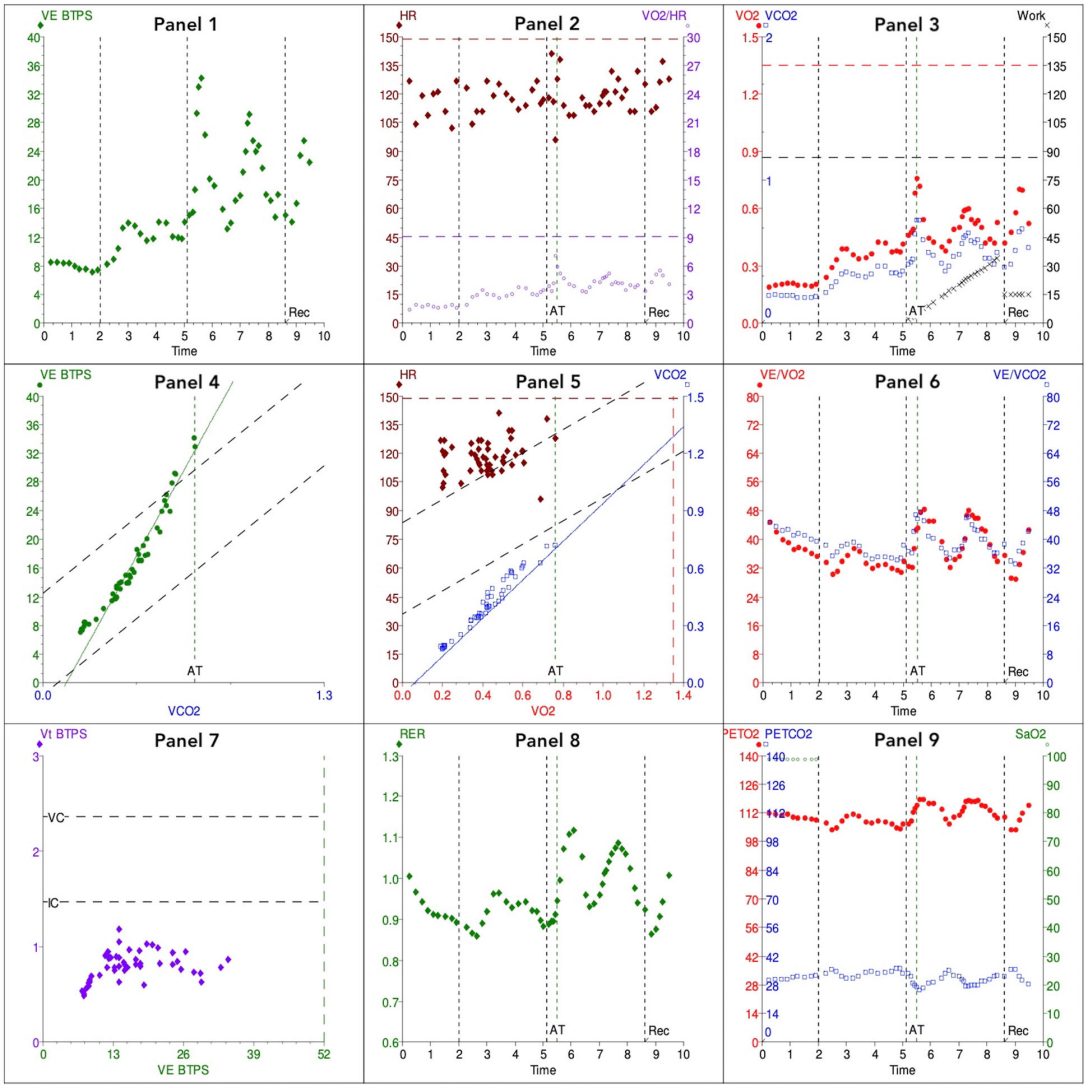
Nine‐panel cardiopulmonary exercise test plot displaying flattened oxygen pulse in panel 2 (purple circles), steep $\dot{V}E/\dot{V}{\mathrm{CO}}_{2}$ slope in panel 4 (green dots), oscillatory ventilation in panels 1 (green diamonds) and 3 (red dots), and high heart rate response in panel 5 (red diamonds). $\dot{V}E/\dot{V}{\mathrm{CO}}_{2}$, ventilatory equivalent for carbon dioxide; $\dot{V}O_{2}$, oxygen consumption; $\dot{V}O_{2}$/HR, oxygen pulse; AT, anaerobic threshold; HR, heart rate; PET CO_2_, end‐tidal partial pressure of carbon dioxide; PET O_2_, end‐tidal partial pressure of oxygen; RER, respiratory exchange ratio; VE BTPS, minute ventilation at body temperature and pressure saturated with water vapour.

Cardiopulmonary exercise testing was conducted with a metabolic cart (MedGraphics, St Paul, MN, USA) and an electromagnetically braked cycle ergometer (Lode, Groningen, Netherlands) with a 10‐W ramp protocol. Resting pulmonary function tests demonstrated moderate air flow obstruction. The test was terminated at 6.5 min due to mouthpiece intolerance and knee pain. She achieved a peak workload of 35 W (40% predicted). She did not reach anaerobic threshold and achieved $\dot{V}O_{2}$ peak of 13.9 ml.kg^−1^.min^−1^ (57% predicted) with an oscillatory ventilation pattern. Her ECG demonstrated atrial fibrillation with an elevated resting heart rate of around 100 beats.min^−1^ but no exercise‐induced ischaemia. Her $\dot{V}E/\dot{V}{\mathrm{CO}}_{2}$ was 53, suggesting significant ventilation‐perfusion mismatching (Fig. 1) and her oxygen uptake efficiency slope of 669 was significantly reduced. The oxygen pulse was flattened consistent with an impaired stroke volume response to exercise.

Despite early termination, CPET suggested significant heart failure evidenced by low $\dot{V}O_{2}$ peak, high $\dot{V}E/\dot{V}{\mathrm{CO}}_{2}$, flattened oxygen pulse, low oxygen uptake efficiency slope and oscillatory ventilation. Surgery was deemed too high risk and she was therefore referred by the CPET anaesthetist to a cardiologist for optimisation. The cardiologist commented on her minimal cardiorespiratory symptoms and considered her as New York Heart Association Class 2, identifying poor heart rate control (resting rate 122 beats.min^−1^) and poor ventricular function. Bisoprolol and enalapril doses were increased and digoxin was added on advice by the cardiology team.

Three months later, 24‐h ECG demonstrated improved heart rate control (mean rate 93 beats.min^−1^). Repeat CA125 was normal (34 kU.l^−1^) and the adnexal mass was unchanged on repeat abdominal computed tomography scan. In view of the risks of surgery and the resolution of cancer markers, the patient and the multidisciplinary team opted for a surveillance approach. Three years later, her gynaecological condition was unchanged and follow up echocardiogram identified near normal left ventricular function (ejection fraction 52%).

## Discussion

This case demonstrates the utility of pre‐operative CPET to identify significant but relatively asymptomatic heart failure. Anaerobic threshold, $\dot{V}O_{2}$ peak and $\dot{V}E/\dot{V}{\mathrm{CO}}_{2}$ are the CPET variables most commonly associated with surgical outcomes according to consensus clinical guidelines developed by the International Prehabilitation and Peri‐operative Exercise Testing Society [5]. Our patient had a peak oxygen uptake < 15 ml.kg^−1^.min^−1^ and $\overset{˙}{V}E/\overset{˙}{V}{\mathrm{CO}}_{2}$ > 34, both markers of increased peri‐operative risk. However, the diagnostic utility of additional CPET metrics, such as the flattened oxygen pulse, markedly reduced oxygen uptake efficiency slope and presence of oscillatory ventilation, prompted the diagnosis of significant heart failure requiring optimisation even though the exercise test was sub‐maximal.

Heart failure is a major peri‐operative risk factor and is present in 2.5–10% of patients having non‐cardiac surgery [2]. It is associated with high postoperative complication rates and a mortality rate of around 10% [2]. Although heart failure has classically been attributed to a limitation of cardiac output, there are non‐cardiac components of the oxygen transport cascade involved, such as endothelial and mitochondrial function, lung ventilation and pulmonary vascular resistance, particularly in heart failure with preserved ejection fraction, which is thought to constitute approximately 50% of all heart failure diagnoses [6]. Cardiopulmonary exercise testing can identify and evaluate all heart failure phenotypes. Peak $\dot{V}O_{2}$ < 14 ml.kg^−1^.min^−1^ (or < 12 ml.kg^−1^.min^−1^ in patients taking β‐blockers) is associated with a poor prognosis and 1‐year mortality rates exceeding 20% [6]. Effort independent CPET variables are also diagnostic in heart failure, such as the $\dot{V}E/\dot{V}{\mathrm{CO}}_{2}$ slope, oxygen uptake efficiency slope and exercise oscillatory ventilation. These variables can be utilised in a multiparametric clinical heart failure stratification [3]. Ventilatory equivalent for carbon dioxide slope is a marker of ventilatory inefficiency and is raised in heart failure due to chronic hyperventilation and increased physiological dead space during exercise. A slope > 36 identifies high mortality risk in heart failure patients, with mortality > 20%, if slope ≥ 45 [7]. Our patient's $\dot{V}E/\dot{V}{\mathrm{CO}}_{2}$ slope was 53 reflecting significant disease. Exercise oscillatory ventilation is irregular breathing characterised by regular cyclic variation of ventilation which can persist throughout exercise or disappear in the final stages, and is common in heart failure patients. It is thought to be due to feedback delays between the chemoreceptors and the ventilatory centres. The presence of exercise oscillatory ventilation is associated with a 1‐year mortality > 20% [7]. Oxygen uptake efficiency slope is defined as the slope of the linear relationship between log_10_ minute ventilation (x‐axis) and $\dot{V}O_{2}$ (y‐axis) measured during an incremental exercise test. It is a marker of cardiorespiratory reserve and the effectiveness of oxygen extraction and utilisation. It is reduced in heart failure and correlates with $\dot{V}O_{2}$ peak even in sub‐maximal tests, with oxygen uptake efficiency slope < 1400 indicating a high mortality risk [7]. The oxygen pulse ($\dot{V}O_{2}$/heart rate) normally increases during exercise and reflects the increase in stroke volume and arterial‐mixed venous oxygen content. A plateau or downward displacement of the oxygen pulse suggests a failure of the stroke volume to increase presuming maximal oxygen extraction in the absence of anaemia or hypoxia.

The comprehensive physiological data provided from CPET can be used to estimate the risk of postoperative mortality and morbidity, facilitate shared decision‐making, triage patients to postoperative critical care, allow optimisation, identify hidden comorbidities, evaluate the effects of neoadjuvant cancer treatments and guide prehabilitation exercise training. However, peri‐operative risk thresholds are likely to be surgery specific, related to the magnitude of the procedure and the associated physiological insult. Other methods of functional capacity assessment exist, such as the 6‐min walk test or shuttle walk tests, but they correlate poorly with CPET and do not have the diagnostic ability of CPET [4]. Systematic reviews of CPET have been critical of the evidence base because of selective reporting of variables, unblinded assessments and poor standardisation of testing and outcomes [8]. Cardiopulmonary exercise testing is expensive, requiring trained personnel, specialised equipment and a dedicated facility.

Carbohydrate antigen 125 is a complex glycoprotein, mainly synthesised by mesothelial cells in the pericardium, pleura or peritoneum, and is best known as a biomarker for ovarian cancer but also correlates with volume overload and is a useful measure of congestion in heart failure [1]. Activation of mesothelial cells in response to increased hydrostatic pressures, mechanical stress and inflammatory cytokine formation because of systemic and pulmonary venous congestion is thought to promote CA125 synthesis [1]. Our patient's high CA125 levels were presumed to be caused by ovarian cancer; however, with heart failure optimisation, her CA125 levels returned to normal. Elevated CA125 levels have been found in two‐thirds of patients with acute heart failure and correlate with the severity of congestion and mortality [1, 9]. Carbohydrate antigen 125 levels < 35 kU.l^−1^ are a prognostic threshold in heart failure with levels below this cutoff associated with the lowest risk of adverse outcomes [1]. However, CA125 is not cardiac‐specific and its upregulation can occur in several benign and neoplastic diseases. Carbohydrate antigen 125 has been shown to be a useful tool to risk stratify, monitor and tailor diuretic therapy with a significantly longer half‐life than natriuretic peptides of 5–12 days providing clinical information regarding the heart failure status in a similar way glycosylated haemoglobin evaluates diabetes management [1]. It is most useful in patients with mainly right‐sided heart failure, heart failure with preserved ejection fraction, severe renal dysfunction and older patients [10]. Contrary to natriuretic peptides, CA125 levels are not influenced by age, renal function, ejection fraction or patient weight and CA125 testing is widely available and low‐cost, making it attractive for use in daily clinical practice where a lack of biomarker exists for assessing congestion.

In conclusion, this case demonstrates the importance of CPET in identifying subclinical heart failure and highlights the role of CA125 as a biomarker in both heart failure and malignancy. Collaborative, multidisciplinary assessment altered the patient's treatment trajectory and resulted in her avoiding high‐risk surgery.
